# Rapidly progressive varicella zoster virus vasculopathy in a chemotherapy- and steroid-immunosuppressed patient with refractory diffuse large B-cell lymphoma: diagnostic and therapeutic challenges

**DOI:** 10.1007/s44313-026-00126-5

**Published:** 2026-03-06

**Authors:** Kyoung Il Min, Gi-June Min, Ki-Seong Eom, Seok-Goo Cho

**Affiliations:** Department of Hematology, College of Medicine, Seoul St. Mary’s Hematology Hospital, The Catholic University of Korea, Banpo-Daero 222, Seocho-Gu, Seoul, Republic of Korea

**Keywords:** Varicella zoster virus vasculopathy, Reversible cerebral vasoconstriction syndrome, Diffuse large B-cell lymphoma, Immunosuppression

## Abstract

Varicella zoster virus (VZV) vasculopathy is a rare but severe neurological complication that frequently results in a reversible cerebral vasoconstriction syndrome (RCVS). Diagnosis is often delayed because early manifestations are nonspecific and radiologic findings often overlap with those of other cerebrovascular disorders. We describe the fatal case of a heavily pretreated 60-year-old man with refractory diffuse large B-cell lymphoma undergoing evaluation for chimeric antigen receptor (CAR) T-cell therapy. The patient developed multifocal intracranial arterial stenosis and an acute infarction during chemotherapy-induced immunosuppression. The initial radiological features suggested RCVS; however, cerebrospinal fluid (CSF) analysis using polymerase chain reaction (PCR) and anti-IgG antibodies confirmed the presence of VZV. Despite intravenous acyclovir and high-dose corticosteroids, the vasculopathy progressed rapidly, resulting in recurrent infarctions, seizures, and death. This case highlights the diagnostic complexity of VZV vasculopathy in immunocompromised patients and underscores the importance of early CSF evaluation, heightened clinical vigilance for opportunistic viral complications, and the potential diagnostic value of vessel wall magnetic resonance imaging (MRI) in differentiating VZV vasculopathy from mimicking conditions such as RCVS.

**Supplementary Information:**

The online version contains supplementary material available at 10.1007/s44313-026-00126-5.

## To the Editor

Varicella zoster virus (VZV) vasculopathy is an uncommon but severe neurological complication that can occur following viral reactivation, particularly in immunocompromised patients with hematologic malignancies or those receiving intensive chemotherapy and corticosteroids [1–3]. The clinical spectrum ranges from transient focal deficits to multifocal infarctions and fatal vascular injury; however, diagnosis is often delayed because early manifestations are nonspecific and radiologic findings often overlap with those of other cerebrovascular disorders [1, 4]. Reversible cerebral vasoconstriction syndrome (RCVS) is a major diagnostic pitfall because acute severe headache, multifocal ischemia, and segmental arterial narrowing on angiography are common in both conditions [4–6]. Because of the rapidly expanding population of heavily pretreated patients with lymphoma, especially those undergoing evaluation for chimeric antigen receptor (CAR) T-cell therapy or receiving other salvage regimens associated with profound immunosuppression, awareness of VZV vasculopathy and its distinction from mimicking conditions, such as RCVS, is increasingly crucial for timely and appropriate patient care [7, 8].

Herein, we describe a fatal case of VZV vasculopathy that could not be initially distinguished from RCVS in a patient with refractory diffuse large B-cell lymphoma, highlighting the diagnostic complexity and need for heightened vigilance in this vulnerable population.

## Case

A 60-year-old man was diagnosed with diffuse large B-cell lymphoma, Ann Arbor stage III, following core needle biopsy of the supraclavicular lymph node. Bone marrow evaluation revealed no lymphoma involvement, and cytogenetic and next-generation sequencing analyses revealed no significant genetic abnormalities. The patient received six cycles of rituximab, cyclophosphamide, doxorubicin, vincristine, and prednisolone (R-CHOP) chemotherapy and achieved an initial partial response (PR); however, the disease relapsed within 2 months. The patient was subsequently enrolled in the OLYMPIA-4 clinical trial and received three cycles of rituximab, gemcitabine, cisplatin, and dexamethasone (R-GDP) as part of the control arm; however, his condition remained refractory. The patient was subsequently referred to our institution for CART-cell therapy as a salvage option. Before apheresis, the patient had developed herpes zoster with optic neuritis, which was managed with antiviral agents and high-dose corticosteroid pulse therapy during neurological admission. After resolution of the acute episode, acyclovir prophylaxis (400 mg orally twice daily) was initiated and continued throughout subsequent salvage chemotherapy. Leukapheresis yielded one bag containing 2.01 × 10^9^ CD3⁺ cells. Polatuzumab, vedotin, bendamustine, and rituximab (Pola-BR) was administered as bridging chemotherapy during CAR T-cell manufacturing. However, the CAR T-cell product was deemed out of specifications and could not be released. As an alternative salvage approach, the patient received one full-dose cycle of dexamethasone, L-asparaginase, ifosfamide, carboplatin, and etoposide (DL-ICE) with the intent of proceeding to allogeneic hematopoietic stem cell transplantation, if at least a partial response was achieved. Because of persistent fever without an identifiable infectious source and progressive disease, the regimen was switched to etoposide, prednisone, vincristine (Oncovin), cyclophosphamide, and doxorubicin (hydroxydaunorubicin) (EPOCH) at 75% dose intensity, along with additional oral prednisone (0.5 mg/kg) for metabolic fever control.

**Fig. 1:**
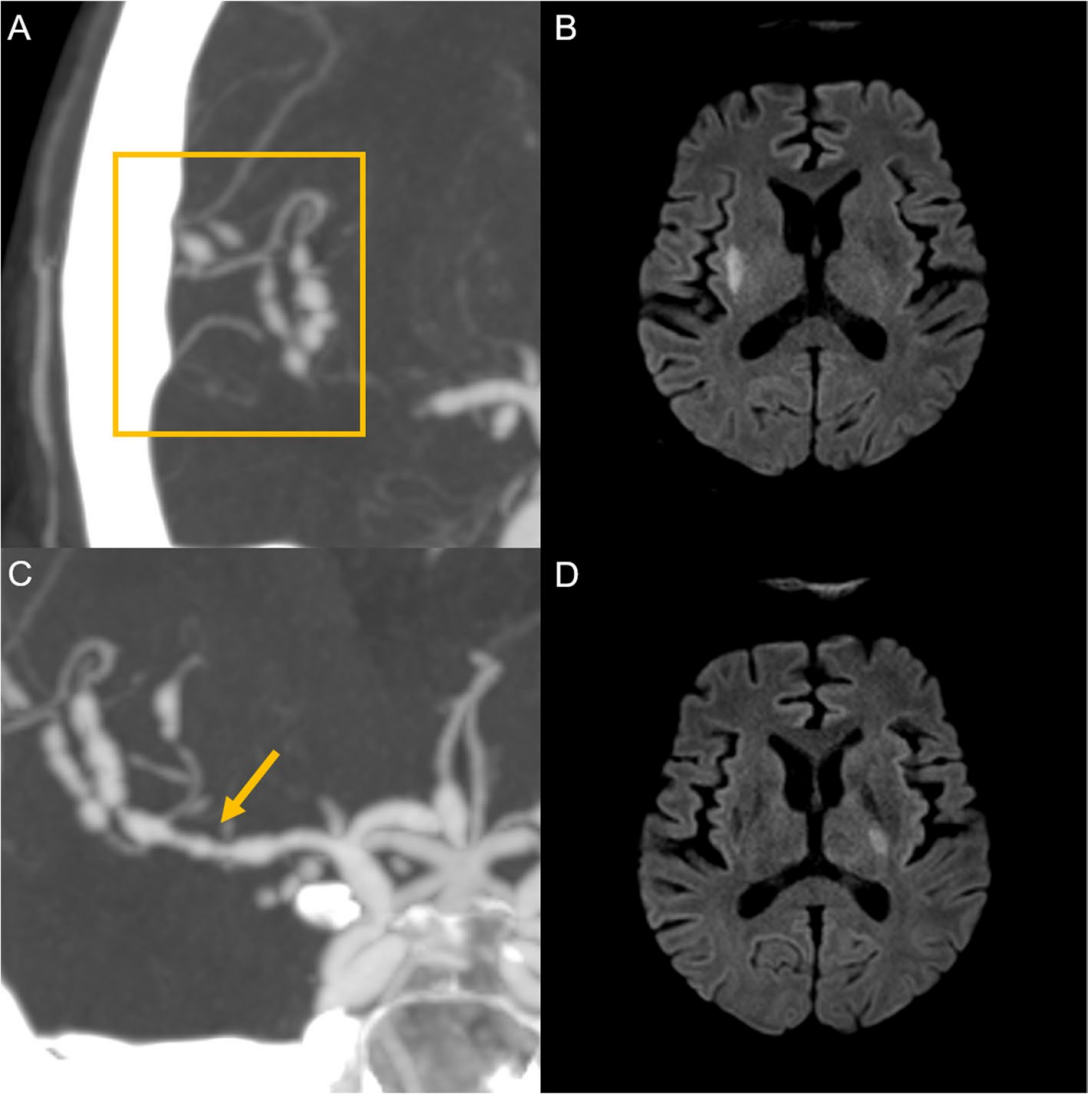
Neuroimaging findings at initial presentation. **A** Initial coronal computed tomography angiography (CTA) showing multifocal “string of bead” appearance of the intracranial arteries (yellow box), which was more pronounced in the right hemisphere. **B** Initial diffusion-weighted magnetic resonance imaging (MRI, b = 1000) showing an acute focal infarction in the right corona radiata. **C** Follow-up coronal CTA reveals newly developed arterial stenosis and dilatation involving the M1 segment of the right middle cerebral artery, indicating disease progression. **D** Follow-up diffusion-weighted MRI (b = 1000) revealing a newly developed acute infarction in the left posterior limb of the internal capsule

During the post-chemotherapy nadir, the patient developed left-sided weakness accompanied by a moderate-intensity headache. Computed tomography angiography (CTA) revealed a multifocal “string of bead” appearance of the intracranial arteries (Fig. 1A), which was more prominent in the right hemisphere. Brain magnetic resonance imaging (MRI) revealed an acute infarction in the right corona radiata (Fig. 1B). Antiplatelet therapy was withheld owing to thrombocytopenia (platelet count, 37 × 10^9^/L), and hydration and atorvastatin therapy (40 mg daily) were initiated. The patient's left arm weakness worsened, and aspirin administration was initiated when the platelet count recovered to > 50 × 10^9^/L after platelet transfusion. Subsequently, a cerebrospinal fluid (CSF) FilmArray® (BioFire Diagnostics, USA) meningitis/encephalitis panel revealed a positive result for VZV, suggesting VZV-associated vasculopathy as the cause of vascular abnormalities and infarction. Notably, herpes zoster-associated optic neuritis occurred approximately 4 months before the onset of cerebrovascular symptoms, with interim clinical stabilization following antiviral therapy and continuous acyclovir prophylaxis. Intravenous acyclovir (10 mg/kg) was administered to treat suspected intracerebral VZV vasculopathy. High-dose intravenous steroid pulse therapy was initiated because corticosteroid use was not contraindicated, such as in active coinfection, and CSF analysis revealed elevated protein levels and inflammatory changes. The patient’s symptoms initially improved, and bispecific antibody treatment was planned. However, follow-up CTA revealed aggravation of the vasculopathy (Fig. 1C), and follow-up brain MRI revealed a newly developed acute infarction in the left posterior limb of the internal capsule (Fig. 1D). Shortly thereafter, the patient developed decreased consciousness and seizures and died several days later.

## Discussion

VZV vasculopathy and RCVS share overlapping radiological and clinical features, often posing a diagnostic challenge [1, 4, 9, 10]. However, their underlying mechanisms differ substantially: VZV vasculopathy results from viral infection and immune-mediated vasculitis, whereas RCVS is a non-inflammatory vasospastic disorder triggered by different stimuli, including vasoactive drugs, postpartum state, and stress [1, 5, 6]. In our patient, pleocytosis in the CSF, positive VZV polymerase chain reaction (PCR) and anti-IgG antibody findings, and segmental stenosis with hemispheric predominance on magnetic resonance angiography were consistent with VZV vasculopathy rather than RCVS. Although both conditions can present with acute headaches and focal neurological deficits, VZV vasculopathy shows persistent vessel stenosis and inflammatory CSF profiles, in contrast to the reversible vasoconstriction and near-normal CSF findings in RCVS. Because the treatment strategies for different conditions differ fundamentally, prompt differentiation is critical for optimizing outcomes. VZV vasculopathy requires antiviral therapy with acyclovir and corticosteroids (acyclovir 10 mg/kg IV for 14 days combined with methylprednisolone 1 mg/kg/day for 5–7 days) [1, 2]. Although adjunctive corticosteroids are recommended for VZV vasculopathy to mitigate virus-associated vascular inflammation, their use in profoundly T-cell-suppressed patients warrants careful risk–benefit assessment, as excessive immunosuppression may theoretically hinder viral clearance. RCVS is primarily managed using calcium channel blockers, such as nimodipine 30–60 mg PO QID or verapamil 80–120 mg PO TID, nonsteroidal anti-inflammatory drugs, and symptomatic care [11]. In this case, RCVS remained the leading differential diagnosis until VZV was identified in the CSF. The initial diagnostic prioritization was driven by the characteristic angiographic findings of multifocal segmental intracranial arterial stenosis and dilatation with hemispheric predominance. Atypical headache features and mild CSF protein elevation were not considered sufficient to exclude RCVS in the context of corticosteroid use and profound immunosuppression, and the absence of cutaneous zoster lesions or cranial neuropathies further lowered the suspicion of VZV vasculopathy. During this period, aspirin was administered for cerebral infarction, along with a statin, an NSAID for headache control, and a calcium channel blocker, while closely monitoring the clinical course. After confirmation of the VZV infection, acyclovir and steroid pulse therapy were initiated, resulting in improvements in focal paralysis and headache. Corticosteroids, previously considered a potential treatment for RCVS, have demonstrated limited efficacy and are associated with adverse outcomes [12, 13]. This case highlights how easily VZV vasculopathy can be mistaken for RCVS, underscoring the need for careful clinical and radiological evaluations in immunocompromised patients (Supplementary Table 1).

In addition to these diagnostic considerations, the patient’s profound immunosuppression increased the susceptibility to severe VZV reactivation and vasculitis. High-dose corticosteroids administered to treat herpes zoster-associated optic neuritis, combined with intensive cytotoxic chemotherapy and CAR T-cell bridging regimens, may have contributed to B-cell aplasia, T-cell suppression, and subsequent CAR T-cell manufacturing failure. Notably, the patient rapidly developed progressive VZV vasculopathy despite continuous acyclovir prophylaxis, representing a breakthrough infection with profound chemotherapy- and steroid-induced immunosuppression. This observation suggests that standard-dose antiviral prophylaxis may be insufficient to prevent severe central nervous system VZV complications in selected high-risk patients. When evaluating patients for CAR T-cell therapy or administering salvage/bridging chemotherapy, clinicians should be vigilant of opportunistic viral vasculopathies that may present atypically and progress rapidly in profoundly immunosuppressed patients. In addition, the interval between leukapheresis, CAR-T cell manufacturing failure, and subsequent salvage regimens represents a particularly vulnerable immunological nadir, during which patients are at a heightened risk of opportunistic viral complications. High-resolution vessel wall MRI, which can detect the inflammatory enhancement characteristics of VZV vasculitis, was not performed. This procedure may have helped to differentiate VZV vasculopathy from RCVS earlier in the disease course. Finally, as herpes virus reactivation is increasingly being recognized in patients treated with CAR-T cells or bispecific antibodies, emerging evidence highlights the potential role of extended antiviral prophylaxis and proactive neurological surveillance in selected high-risk populations [7, 14].

These observations emphasize that VZV vasculopathy requires rapid recognition, particularly in profoundly immunosuppressed patients. The clinical implications include early CSF evaluation, consideration of vessel wall MRI, and proactive antiviral strategies for CAR T cell candidates. Updated prophylactic approaches and standardized neurological surveillance may help prevent catastrophic outcomes in this growing population, as herpes virus reactivation has been increasingly reported after CAR T cell therapy. In summary, this case highlights the critical need to recognize VZV vasculopathy early in patients with profoundly immunosuppressed lymphoma, as timely differentiation from RCVS can be lifesaving.

## Supplementary Information

Supplementary Material 1.

## Data Availability

No datasets were generated or analyzed during the current study.
