# Supplementary material for: Rapidly progressive varicella zoster virus vasculopathy in a chemotherapy- and steroid-immunosuppressed patient with refractory diffuse large B-cell lymphoma: diagnostic and therapeutic challenges
Source: Blood Res. 2026 Mar 6;61(1):11. doi: 10.1007/s44313-026-00126-5 (PMC12965949; doi:10.1007/s44313-026-00126-5)
Supplement: Supplementary file 1 — Supplementary Material 1. [file 44313_2026_126_MOESM1_ESM.docx]

**Supplementary Material**

**Article Title:** Rapidly Progressive Varicella Zoster Virus Vasculopathy in a Chemotherapy- and Steroid-Immunosuppressed Patient with Refractory Diffuse Large B-cell Lymphoma: Diagnostic and Therapeutic Challenges

**Journal Name:** Blood Research

**Author names:** Kyoung Il Min, Ki-Seong Eom, Seok-Goo Cho, and Gi-June Min*

**Affiliations**: Department of Hematology, Seoul St. Mary's Hematology Hospital, College of Medicine, The Catholic University of Korea, Banpo-daero 222, Seocho-Gu, Seoul, Republic of Korea

**Corresponding author:**

**Gi-June Min, M.D., Ph.D.**

Department of Internal Medicine, College of Medicine, Seoul St. Mary's Hematology Hospital, The Catholic University of Korea, Banpo-daero 222, Seocho-Gu, Seoul 137-701, Republic of Korea

E-mail: [beichest@catholic.ac.kr](mailto:beichest@catholic.ac.kr)

**Supplementary Table 1. Key distinguishing clinical, laboratory, and imaging features of VZV vasculopathy versus reversible cerebral vasoconstriction syndrome (RCVS)**

| Category | VZV Vasculopathy  *(Varicella-Zoster Virus Vasculopathy)* | RCVS  *(Reversible Cerebral Vasoconstriction Syndrome)* |
| --- | --- | --- |
| Primary Cause | Viral invasion of cerebral vessels + immune-mediated vasculitis | Non-inflammatory vasospasm, often triggered by vasoactive agents (SSRIs, triptans, sympathomimetics), postpartum state, or acute stress |
| Pathogenesis | Reactivation of VZV in cranial nerves or ganglia → viral spread to cerebral arteries → vessel wall inflammation, necrosis, and thrombosis | Transient dysregulation of cerebral vascular tone → multifocal vasoconstriction without inflammation |
| Clinical Presentation | Acute headache + focal neurologic deficits (e.g., hemiparesis, cranial neuropathy, visual disturbances) | Sudden, severe “thunderclap headache” ± transient focal neurologic symptoms |
| CSF Findings | Inflammatory profile: pleocytosis, ↑ protein; positive VZV PCR and/or anti-VZV IgG | Typically, normal or mild protein elevation; no pleocytosis |
| MRI/MRA | Multifocal stenosis and dilatation (steno-dilatation), ischemic lesions, often hemispheric predominance | Bilateral, multifocal vasoconstriction; reversible within 2–3 months |
| Diagnostic Evidence | Detection of VZV DNA in CSF or presence of anti-VZV IgG antibody in CSF | Clinical + radiologic findings showing reversible vasoconstriction |
| Treatment | Acyclovir + high-dose corticosteroids (inflammatory vasculitis) | Nimodipine, antihypertensives, analgesics; corticosteroids generally not recommended |
| Prognosis | Risk of recurrent stroke, neurologic sequelae, and death if untreated | Typically, complete recovery within 2–3 months; relapse uncommon |

**Abbreviations:** VZV, varicella-zoster virus; RCVS, reversible cerebral vasoconstriction syndrome; CSF, cerebrospinal fluid; PCR, polymerase chain reaction; MRI, magnetic resonance imaging; MRA, magnetic resonance angiography; MCA, middle cerebral artery; NSAIDs, nonsteroidal anti-inflammatory drug; SSRIs, selective
